# Nonprofit human milk banking: On a challenging path to global equity

**DOI:** 10.1111/mcn.13623

**Published:** 2024-01-10

**Authors:** Natalie S. Shenker, Sushma Nangia

**Affiliations:** ^1^ Department of Surgery and Cancer Imperial College London London UK; ^2^ Human Milk Foundation Rothamsted Institute Hertfordshire UK; ^3^ Vatsalya Maatri Amrit Kosh National Comprehensive Lactation Management Centre New Delhi Delhi India; ^4^ Department of Neonatology Lady Hardinge Medical College & Kalawati Saran Children's Hospital New Delhi Delhi India

Human milk banks provide a vital clinical service for low‐birthweight and sick infants and their mothers. Where used to feed preterm infants, access to screened donor human milk (DHM) reduces the incidence and severity of necrotising enterocolitis, need for parenteral nutrition and the duration of hospital stay (Quigley et al., 2018). Where used in a setting of optimal lactation support with kangaroo care and Baby Friendly Hospital Initiative accreditation (Mondkar et al., 2022), maternal breastfeeding and psychological wellbeing can be supported (Brown & Shenker, 2022), giving the infant and mother the best chance of good long‐term outcomes. Recent World Health Organization (WHO) ‘Recommendations on the care of preterm or low‐birthweight infants’ recommend with moderate certainty that DHM should be available for all preterm infants where there is insufficient maternal milk (World Health Organization, 2022).

In the last 3 years, milk banking services have been challenged globally as a result of the COVID‐19 pandemic (Bhasin et al., 2022; Shenker et al., 2021), in part as a result of a lack of broader awareness of the sector in public health considerations. This special supplement highlights themes that arose from the International Expert Meeting on the Donation and Use of Human Milk, hosted by Zurich University, including the historical underpinning of current service vulnerabilities, technical advances in the context of a relative lack of research and innovation funding (Weaver, G. and Chatzixiros 2023), global inequalities in provision (Israel‐Ballard and Manson 2023), and the ethical considerations of prioritising a rationed product of human origin (Herson and Weaver 2023).

Milk banking continues to face challenges. Few countries have a comprehensive system of equitable and assured DHM provision, leaving almost a million low‐birthweight infants without access to DHM in the context of insufficient maternal milk in both high and low‐resource settings (Shenker et al., 2020). Furthermore, increasing demand is driving a market. In both wealthy and low‐resource countries, the commercialisation of human milk provision could further weaken the essential links between DHM provision and lactation support (Reimers & Coutsoudis, 2021), exacerbated by the absence of regulations that can reduce the risk of exploitation of both milk providers, recipients and health care services (Klotz et al., 2021; Shenker et al., 2023). In the absence of regulation, for‐profit companies are adopting many of the strategies of the formula industry to create the market for their products (Newman & Nahman, 2020). Reported techniques include marketing reps working directly with neonatal intensive care unit teams, industry‐funded trials showing benefit unsupported by independent data, industry limiting access to their products for trials over which they have no control, industry funding essay prizes and training for junior neonatologists and companies contacting mothers directly through social media platforms.

Increased commercialisation may represent the start of a process that leads to the exploitation of providers and their infants, particularly the vulnerable and bereaved, while introducing safety concerns, the absence of embedded provision within optimal lactation support services, and a drive towards access on the basis of capacity to pay. Liquid human milk products replace maternal milk volume, but the WHO Code on the marketing of breast milk substitutes does not include human milk products as breast milk substitutes, leaving families and doctors vulnerable to similar strategies used effectively by the infant formula industry (Clark & Adhanom Ghebreyesus, 2022; Newman & Nahman, 2020; Pérez‐Escamilla et al., 2023; Rollins et al., 2023).

Unregulated commercialisation risks human milk being even further limited in supply, particularly to those infants and families who would benefit most through health or socioeconomic factors. Individual countries, including Cambodia and India, have already introduced laws to limit the sale and/or export of milk. The Cambodian Minister for Health, when announcing the legislation, noted ‘Even though we are still poor, we are not so poor that we have to sell human breast milk’ (Independent, 2017). This approach now needs to be replicated by policymakers wherever regulatory gaps exist.

Although the International Expert Meeting concluded with an acknowledgement of challenges created by current gaps in regulation, minimum standards and research, there is now renewed hope within the milk banking sector. Since the global milk bank community came together at the start of the pandemic and launched a call to action for increased investment in milk banking (Shenker et al., 2020), the WHO have convened a Guideline Development Group, the European Union has included human milk in the revised Blood Tissues and Cells Directive, and legislation is currently proposed in the United States to ensure equity in DHM provision. At this critical point in the development of global human milk bank services, an increasing onus is now on governments along with country and regional‐level regulatory agencies to ensure equitable milk banking services are implemented and strengthened to safeguard and support breastfeeding, mothers and infants.
